# Overall effects of microplastics on brain

**DOI:** 10.3389/ftox.2025.1619096

**Published:** 2025-11-20

**Authors:** Su-jun Fang, Zhao-di Yin, Li-fan Li, Qi Cai, Peng-fei Zheng, Li-zhen Chen

**Affiliations:** 1 Department of Pharmacy, The First Hospital of Putian City, Putian University, Fujian, China; 2 College of Environmental and Biological Engineering, Putian University, Fujian, China; 3 Department of Neurology, Peking University People’s Hospital, Beijing, China; 4 School of Medicine, Nankai University, Tianjin, China; 5 School of Computer Science and Technology, University of Science and Technology of China, Anhui, China

**Keywords:** microplastics, nanoplastics, brain, neurotoxicity, blood-brain

## Abstract

Microplastic (MP) and nanoplastic (NP) pollution represents a pervasive environmental issue, raising significant concerns regarding potential neurotoxicity and impacts on brain health. This review synthesizes recent research findings to provide a comprehensive overview of the effects of MPs/NPs on the brain. Evidence demonstrates that MPs/NPs can cross critical biological barriers, including the blood-brain barrier and the placenta, gaining access to the central nervous system (CNS) and the developing fetal brain, influenced by particle size, charge, and the biomolecular corona. Once present, MPs/NPs trigger multiple detrimental pathways, including oxidative stress, persistent neuroinflammation involving microglia and astrocytes, mitochondrial dysfunction leading to energy deficits, disruption of crucial neurotransmitter systems, and direct neuronal damage. Critically, NPs have been shown to promote the aggregation of proteins implicated in neurodegeneration, such as alpha-synuclein. These mechanistic disturbances translate into observable adverse outcomes in experimental models, ranging from cognitive impairments in learning and memory to behavioral abnormalities and pathologies resembling human neurodegenerative and neurodevelopmental disorders. Toxicity is modulated by particle characteristics, co-exposures, and host factors like age and sex, with indirect effects via the gut-brain axis also playing a significant role. While current evidence, primarily from animal models often using high doses, strongly indicates a neurotoxic potential, significant research gaps remain concerning human risk assessment under chronic, low-level environmental exposure conditions and the effects of environmentally aged, mixed-plastic particles. Future research should prioritize human studies, environmentally realistic exposure scenarios, and differentiating direct versus indirect neurotoxic mechanisms to accurately evaluate the threat MPs/NPs pose to human brain health.

## 1 Introduction

Microplastics (MPs), defined as plastic particles less than 5 mm in size, represent a pressing environmental concern due to their prevalence in terrestrial and aquatic ecosystems (Cottom et al., 2024). The discharge of MPs is estimated to be in the millions of metric tons annually, primarily sourced from rivers, which underscores their widespread distribution and the insufficiency of existing oceanic assessments that capture only a fraction of this pollution (Weiss et al., 2021). In healthcare, various medical practices contribute significantly to microplastic generation, primarily through single-use items and equipment disposal (Shelton et al., 2022). These MPs pose various environmental risks, including the potential to harm aquatic life and disrupt food chains, thereby causing cascading effects in ecosystems and human health (Marfella et al., 2024). The increasing recognition of microplastic pollution has enhanced the urgency of addressing its sources and mitigating its impact on the environment.

Evidence indicates that exposure to MPs can induce neurotoxicity characterized by oxidative stress and inflammatory responses, thereby disrupting neurotransmission and impacting behavioral outcomes (Ye et al., 2023; Marcellus et al., 2024; Sönmez, 2024) (Table 1). The prolonged exposure to MPs results in significant neurotoxicity, which can implicate long-term neurobiological effects, including possible cognitive deficits (Wu Y. et al., 2023). Additionally, studies highlight the capacity of MPs to affect neurotransmitter systems crucial for cognitive functioning, which can manifest in altered behavior patterns in various aquatic models (Sönmez, 2024; Chen et al., 2024). These particles disrupt neurobiological pathways, and their ability to act as vectors for additional toxicants can exacerbate neurotoxic effects (Alvítez et al., 2025).

**TABLE 1:** Summary of neurological effects of microplastics (MPs) and nanoplastics (NPs) in animal models.

Microplastic	Model	Key neurological/behavioral outcomes	References
Polystyrene Nanoplastics (PS-NPs)	Mice	Accelerated α-synuclein aggregation; PD-like motor dysfunction; Neuroinflammation and oxidative stress	Liang et al. (2025)
Microplastics (low-dose)	Mice, *C. elegans*	Accelerated dopamine neuron degeneration; Induced PD-like pathology via gut barrier disruption	Bai et al. (2025)
Polystyrene Microplastics (PS-MPs)	Mice (AD model)	Promoted cognitive impairment; Aggravated neuroinflammation by inducing microglial pyroptosis	Wang et al. (2024a)
0.5 µm Polystyrene Microplastics	Velvet Swimming Crab	Demonstrated microplastic retention in brain tissue for at least 21 days	Crooks et al. (2019)
Polylactic Acid (PLA) Microplastics	Zebrafish	Induced sluggish behavior and neuron loss; Disrupted the microbiota-gut-brain axis, toxicity enhanced by aging	Qian et al. (2025)
Amino-modified PS-NPs	Mice	Disrupted blood-brain barrier (BBB) integrity; Induced neuronal apoptosis and Alzheimer’s disease-like neurotoxicity (Ac-Tau formation)	Bai et al. (2024)
PS-NPs (50 nm)	Mice	Caused PD-like neurodegeneration by inducing energy metabolism disorders in the brain; Reduced ATP content in substantia nigra and striatum	Liang et al. (2022)
PS-MPs and Mercury	European Seabass	Caused neurotoxicity via acetylcholinesterase (AChE) inhibition; Increased oxidative damage in the brain	Barboza et al. (2023)
PS-NPs	Zebrafish (males)	Induced Parkinson’s-like behavior	Xian et al. (2024)
Polyethylene (PE) Microplastics	African Catfish	Altered neurological biomarker enzymes (AChE, MAO); Caused neuronal degradation and necrosis in the telencephalon and cerebellum	Hamed et al. (2022b)
PS-MPs	Mice	Impaired learning and memory; Caused neuroinflammation in the hippocampus via the vagus nerve-dependent gut-brain axis	Lee et al. (2022)
PS-MPs (0.5, 4, 10 µm)	Mice	Accumulated in the brain, disrupting the BBB; Reduced dendritic spine density and induced cognitive/memory deficits	Zhou et al. (2022)
1–5 μm MPs (PS, PMMA)	Honey Bee	Impaired learning and memory; Penetrated the blood-brain barrier and accumulated in the brain	Pasq et al. (2024)
PS-NPs (80 nm)	Zebrafish	Induced learning and memory deficits; Caused oxidative damage and aggravated brain aging markers	Zho et al. (2023)
PS-NPs (30–50 nm)	Mice	Induced cognitive dysfunction (spatial and fear memory); Activated microglia, leading to neuroinflammatory responses	Paing et al. (2024)

Considering the rising prevalence of MPs in the environment, their potential connections to neurodegenerative disorders and cognitive deficiencies underscore an urgent need for comprehensive investigations into their long-term health implications. Therefore, this review aims to describe the latest research to comprehensively summarize the impact of MPs on brain health.

## 2 Microplastic/nanoplastic access to the brain

The potential for MPs and nanoplastics (NPs) to exert neurotoxic effects hinges critically on their ability to reach the central nervous system (CNS). The capacity of these synthetic materials to infiltrate the highly protected environment of the brain raises significant concerns regarding their potential contribution to neurological dysfunction, cognitive impairment, and neurodegenerative disease initiation or progression (Zheng et al., 2024). The primary routes implicated involve direct penetration of the blood-brain barrier (BBB), potential translocation via the olfactory system, and possibly entry following inhalation, alongside indirect influences modulated by the gut-brain axis.

The BBB represents the most extensively studied portal for MP/NP entry into the brain parenchyma. Research indicates that NPs, following oral administration in animal models, can be rapidly absorbed into the bloodstream and subsequently detected within brain tissues, demonstrating their capacity to traverse the BBB (Yang et al., 2022; Kopatz et al., 2023). Studies utilizing polystyrene NPs have shown accumulation in the cerebrum and cerebellum shortly after exposure (Yang ZS. et al., 2022; Shan et al., 2022). This passage is not merely theoretical; specific types of MPs and NPs, including polyethylene, polystyrene, polypropylene, and polyvinyl chloride, have been identified in human cerebrospinal fluid, providing direct evidence of CNS access in humans (Xie et al., 2024). The mechanisms facilitating this translocation appear multifaceted. Amino-modified polystyrene NPs (APS-NPs) have been shown to disrupt the integrity of the BBB by interfering with tight junction proteins, an effect potentially mediated through the TLR2/MMP9 axis (Bai et al., 2024). Furthermore, studies suggest that NPs can utilize cellular transport mechanisms, such as endocytosis and macropinocytosis within endothelial cells, to gain entry into the brain (Ma et al., 2024). Transcytosis across the endothelial layer has also been implicated, allowing particles to move from the bloodstream into the brain tissue (Cho et al., 2024). Notably, inflammatory conditions can exacerbate BBB permeability, leading to enhanced uptake and transport of MPs/NPs into the brain, suggesting a potential vulnerability factor in individuals with pre-existing inflammation (Cho et al., 2024).

The physicochemical characteristics of the particles themselves play a crucial role in modulating their ability to penetrate the BBB. Particle size is a significant determinant, with considerable evidence indicating that NPs (<100 nm or sometimes defined up to 1,000 nm, Zhao et al. (2023a). possess a greater capacity for BBB translocation compared to larger MPs (Yang ZS. et al., 2022; Waring et al., 2018). Direct comparisons have shown higher uptake and transendothelial transport rates for smaller polystyrene particles (e.g., 0.2 µm) compared to larger ones (e.g., 1.0 µm) in BBB models (Cho et al., 2024). However, defining NPs solely based on engineering size standards may be insufficient, and their ability to cross biological barriers should perhaps be a defining characteristic (Zhao XL. et al., 2023). Surface properties, including charge and chemical modifications, also significantly influence brain access. For instance, positively charged NPs have been observed to accumulate readily in the brain (Teng et al., 2022), and specific surface modifications can alter the route or efficiency of endothelial cell entry (Ma et al., 2024).

The interaction of MPs/NPs with biological molecules upon entering the bloodstream, leading to the formation of a “biomolecular corona,” adds another layer of complexity to BBB transport. This corona, composed of proteins and lipids, modifies the particle’s surface identity and influences its biological interactions. Intriguing, yet potentially conflicting, findings exist regarding the corona’s role. One study using computational modeling and experimental data suggested that specific corona components could differentially affect BBB passage; cholesterol molecules adsorbed onto NPs were proposed to enhance membrane interaction and facilitate passive transport across the BBB, whereas certain protein coronas appeared to inhibit this process (Kopatz et al., 2023). Conversely, another investigation concluded that the formation of a biomolecular corona around polystyrene and polyvinyl chloride NPs reduced their overall ability to permeate an *in vitro* BBB model by decreasing cellular uptake and transcytosis (Monikh et al., 2024). This discrepancy highlights the intricate nature of corona-mediated transport and suggests that the net effect likely depends on the specific composition of the corona (influenced by particle properties and the biological milieu) and the precise mechanisms governing transport across different BBB models. Evidence from maternal exposure studies further underscores the potential for NPs to reach the fetal brain. NPs ingested by pregnant animals have been shown to cross both the intestinal and placental barriers, entering fetal circulation and subsequently detected in various fetal organs, including the brain (Cary et al., 2023; Wang et al., 2025; Dibbon et al., 2024; Harvey et al., 2023). This transplacental transfer route represents a critical window of vulnerability for neurodevelopmental toxicity.

While the BBB is a major focus, alternative pathways for MP/NP entry into the CNS warrant consideration. The olfactory pathway presents a potential direct route from the nasal cavity to the brain, bypassing the BBB. Supporting this, MPs have been detected in human olfactory bulb tissue post-mortem, suggesting that inhaled or otherwise nasally deposited particles could translocate along olfactory nerves (Amato et al., 2024). Inhalation exposure is increasingly recognized as a relevant route for MP/NP entry into the body. Studies indicate that inhaled MNPs can lead to neuronal oxidative stress, cytotoxicity, and neurodegeneration, implying translocation from the lungs to the brain, although the precise mechanisms (e.g., systemic circulation following alveolar absorption, or direct neural pathways) require further elucidation (Vojnits et al., 2024; Kang et al., 2024; Xia et al., 2024).

Furthermore, the integrity of the gut barrier and the composition of the gut microbiota, collectively known as the gut-brain axis, can indirectly influence brain health and potentially modulate MP/NP access or neurotoxicity. Several studies highlight that MP/NP exposure disrupts gut barrier function (“leaky gut”) and induces gut dysbiosis (Zheng et al., 2024; Liang et al., 2025; Bai et al., 2025; Shi et al., 2024; Chen et al., 2023; Jin et al., 2019). While primarily mediating toxicity through inflammatory signaling or altered metabolite production affecting the brain, a compromised gut barrier could theoretically increase the systemic load of MPs/NPs or associated inflammatory mediators, indirectly facilitating their subsequent access to or impact on the CNS. For instance, disruption of the intestinal barrier has been explicitly linked to subsequent BBB damage and neuroinflammation following MP exposure (Yin et al., 2022a; Jin et al., 2022). Therefore, while not a direct route of physical entry to the brain itself, the gut-brain axis represents a critical interface influencing the systemic conditions that may permit or exacerbate MP/NP passage across the BBB.

## 3 Mechanisms of microplastic/nanoplastic neurotoxicity

Once MPs and NPs gain access to the CNS, either through direct penetration of protective barriers or indirectly via systemic circulation following ingestion or inhalation, they initiate a complex cascade of detrimental molecular and cellular events.

Beyond the well-documented downstream effects of oxidative stress and neuroinflammation, a more fundamental mechanism of neurotoxicity appears to originate from the physical presence and processing of NP within the neuron itself. Once NPs traverse the cell membrane via endocytic pathways, they are trafficked to lysosomes for degradation. However, because they are indigestible by lysosomal enzymes, NPs accumulate, leading to a critical disruption of the autophagic flux - the cell’s essential housekeeping process for clearing cellular debris and damaged organelles. This interruption of autophagy is a pivotal upstream event, or the “fire,” that subsequently triggers a cascade of cytotoxic consequences. The resulting lysosomal damage can lead to endoplasmic reticulum stress, mitochondrial instability, and the release of lytic enzymes, which in turn generate the “smoke” of oxidative stress and pro-inflammatory reactions. This core disruption of cellular proteostasis also provides a direct link to neurodegeneration, as the failure of cellular cleaning mechanisms can promote the aggregation of toxic proteins and interfere with the ubiquitin-proteasome system (Casella and Ballaz, 2024).

Oxidative stress emerges as a central and frequently reported mechanism of MP/NP-induced neurotoxicity. Exposure to various MPs and NPs, particularly polystyrene, has been shown to disrupt the delicate balance between pro-oxidants and antioxidants within brain tissue (Zheng et al., 2024). This involves the excessive generation of reactive oxygen species (ROS), leading to cellular damage (Umamaheswari et al., 2021; Zhao KM. et al., 2023). Concurrently, studies report alterations in the brain’s antioxidant defense systems; activities of key enzymes such as superoxide dismutase (SOD), catalase (CAT), and glutathione peroxidase (GPx) are often perturbed, and levels of crucial non-enzymatic antioxidants like glutathione (GSH) are frequently depleted (Yang J. et al., 2023; Wang SW. et al., 2022; Kamel et al., 2025; Hamed et al., 2022a). This redox imbalance results in oxidative damage to vital biomolecules, evidenced by increased lipid peroxidation, often measured by elevated malondialdehyde (MDA) levels in brain tissue (Hoyo-Alvarez et al., 2022; Sarasamma et al., 2020; Barboza et al., 2018). This cascade of oxidative damage is implicated not only in direct cellular injury but also as a trigger for subsequent inflammatory and apoptotic pathways within the CNS (Bai et al., 2025; Umamaheswari et al., 2021).

Neuroinflammation is another prominent mechanism intricately linked to MP/NP exposure. The presence of these foreign particles within the brain parenchyma acts as a potent stimulus for the activation of resident immune cells, primarily microglia and astrocytes (Shan et al., 2022; Li et al., 2024; Adamiak et al., 2025). Activated microglia and astrocytes release a battery of pro-inflammatory mediators, including cytokines such as tumor necrosis factor-alpha (TNF-α), interleukin-1 beta (IL-1β), and interleukin-6 (IL-6) (Liang et al., 2025; Kwon et al., 2022; Zheng et al., 2023; Paing et al., 2024). Specific inflammatory signaling pathways, such as the Toll-like receptor (TLR)/MyD88/NF-κB pathway and the NLRP3 inflammasome pathway, are triggered by MP/NP exposure, leading to sustained inflammation and cellular stress (Xia et al., 2024; Kwon et al., 2022; Yin et al., 2022b; Liu et al., 2025). This neuroinflammatory milieu contributes significantly to neuronal dysfunction and death, potentially through mechanisms like pyroptosis (programmed cell death associated with inflammation (Wang GH. et al., 2024), or excitotoxicity secondary to impaired glial function (Adamiak et al., 2025; Su et al., 2025). The inflammatory response can also further compromise BBB integrity, potentially creating a vicious cycle of particle influx and inflammation (Yin et al., 2022a; Jin et al., 2022).

Mitochondrial dysfunction represents a critical hub in MP/NP-induced neurotoxicity, impacting cellular energy homeostasis and survival (Wu et al., 2025; Yao et al., 2023). Studies show that NPs can localize within neuronal mitochondria following cellular uptake (Ma et al., 2024; Tang et al., 2022). This accumulation is associated with significant impairment of mitochondrial function, including disruption of the electron transport chain (specifically complex I inhibition is suggested (Huang et al., 2023)), reduced mitochondrial membrane potential, and a consequent decline in ATP production (Liang et al., 2022; Yang S. et al., 2023). This energy deficit can severely compromise neuronal function and viability. Furthermore, mitochondrial damage can trigger pathways like excessive mitophagy (the selective degradation of mitochondria by autophagy), potentially mediated via the AMPK/ULK1 pathway, which, while initially protective, can lead to cell death if overwhelmed (Tang et al., 2022; Huang et al., 2023). Mitochondrial dysfunction also contributes significantly to oxidative stress through increased ROS generation, linking this mechanism back to the redox imbalance observed (Félix et al., 2023; Jeong et al., 2024). The resultant energy crisis and oxidative damage make neurons particularly vulnerable to degeneration (Gettings et al., 2024).

Direct neuronal damage and the disruption of synaptic function are downstream consequences of the aforementioned mechanisms, as well as potentially direct effects of particle interaction. Histopathological studies reveal neuronal damage, including necrosis, degeneration, altered morphology, reduced dendritic spine density, and cell loss in brain regions like the hippocampus and cerebellum following MP/NP exposure (Jin et al., 2022; Wang SW. et al., 2022; Paing et al., 2024; Yin et al., 2022b; Hamed et al., 2022b). Crucially, MP/NP exposure disrupts neurotransmitter systems essential for brain function. Inhibition of acetylcholinesterase (AChE) activity is a frequently reported finding, suggesting impaired cholinergic transmission (Umamaheswari et al., 2021; Barboza et al., 2023; Liu et al., 2024). Alterations in other neurotransmitter systems, including dopamine, serotonin, glutamate, and GABA, are also documented, contributing to observed behavioral abnormalities (Hoyo-Alvarez et al., 2022; Huang et al., 2022; Torres-Ruiz et al., 2023; Hwang et al., 2022; Han et al., 2023). Furthermore, MPs/NPs interfere with synaptic plasticity mechanisms, notably by inhibiting the CREB/BDNF signaling pathway, which is crucial for learning and memory (Wang SW. et al., 2022). Modifications to key proteins involved in neuronal structure and function, such as Tau protein hyperphosphorylation or acetylation, have also been observed, linking MP exposure to cytoskeletal abnormalities implicated in neurodegeneration (Bai et al., 2024).

A particularly concerning mechanism highlighted is the capacity of MPs/NPs to interact with and promote the aggregation of proteins central to neurodegenerative diseases. Studies specifically demonstrate that polystyrene NPs can accelerate the amyloid aggregation of alpha-synuclein, a protein intrinsically linked to Parkinson’s disease (PD) pathology (Liang et al., 2025; Liang XF. et al., 2024; Ghosal et al., 2024). This interaction appears mediated by hydrophobic forces and results in the formation of oligomeric species with enhanced neurotoxicity (Liang XF. et al., 2024). MPs/NPs may therefore act as seeding agents or scaffolds, facilitating the misfolding and aggregation process. This mechanism provides a direct molecular link between plastic particle exposure and the potential initiation or acceleration of synucleinopathies like PD (Liang et al., 2024). While less explored for other proteins like amyloid-beta in the provided literature, the principle suggests a potential general mechanism for exacerbating proteopathies.

Beyond direct actions within the CNS, indirect mechanisms mediated via peripheral systems, primarily the gut-brain and potentially the lung-brain axis, contribute significantly to neurotoxicity. As established, MPs/NPs disrupt gut microbiota composition and impair intestinal barrier integrity (Chen et al., 2023; Huang et al., 2022; Qian et al., 2025; Wu Z. et al., 2023). This gut dysbiosis and increased intestinal permeability (“leaky gut”) can lead to systemic inflammation and the release of bacterial components like lipopolysaccharide (LPS) into circulation (Bai et al., 2025; Kuai et al., 2024). These systemic inflammatory signals can cross the BBB or activate brain immune cells, contributing to neuroinflammation and neuronal dysfunction even without the physical presence of particles in the brain (Shi et al., 2024; Liang et al., 2024c; Yang QY. et al., 2023). Altered gut metabolite production due to dysbiosis can also impact brain neurotransmitter levels and function (Chen et al., 2023; Huang et al., 2022). Similarly, inhalation exposure leading to lung inflammation can trigger systemic inflammatory responses or potentially allow translocation of inflammatory mediators or even particles themselves (via lung-brain axis) to affect the brain (Kang et al., 2024). These axis-mediated pathways underscore that neurotoxic effects can arise even from exposures where significant direct particle translocation to the brain is limited.

Alpha-synuclein fibrillation is nucleated by anionic nanoplastics through specific binding to its non‐amyloid component (NAC) region, markedly shortening lag times and enhancing fibril elongation (Liu et al., 2023). Concurrently, adsorption of α-synuclein onto polystyrene nanoplastics forms a dynamic protein corona that destabilizes the native helical structure, exposing hydrophobic patches and stabilizing β-sheet nuclei (Somarathne et al., 2024). This interfacial catalysis lowers the thermodynamic barrier for amyloid assembly, driving rapid precipitation of insoluble aggregates reminiscent of Lewy bodies (Liu et al., 2023; Somarathne et al., 2024). Such heterogeneous nucleation surfaces concentrate monomeric α-synuclein and promote cross–β-sheet stacking, suggesting environmental nanoplastic exposure could exacerbate Parkinsonian proteinopathies.

The literature paints a complex picture of MP/NP neurotoxicity, implicating a web of interconnected cellular and molecular mechanisms. Oxidative stress, neuroinflammation, and mitochondrial dysfunction appear as core, frequently co-occurring pillars of toxicity. However, the precise initiating events and the hierarchical relationship between these pathways remain incompletely resolved. Does particle interaction primarily trigger ROS production, which then drives inflammation and mitochondrial damage? Or does inflammation initiated by microglial recognition of the foreign particle lead to secondary oxidative stress and metabolic disruption? Different studies implicitly support varying sequences, likely influenced by particle type, dose, exposure duration, and the specific brain region or cell type examined. Furthermore, the relative contribution of direct particle-cell interactions within the brain versus indirect effects mediated by systemic inflammation originating from the gut or lungs is a critical question. While direct translocation and accumulation in the brain are clearly demonstrated, especially for NPs, the functional consequences observed sometimes appear disproportionate to the seemingly low levels of accumulated particles reported in some studies. This suggests that indirect, systemically mediated mechanisms, possibly involving persistent low-grade peripheral inflammation or altered gut metabolite signaling impacting the brain via the gut-brain axis, might play a more substantial role than currently appreciated, or at least synergize potently with direct particle effects. The finding that alpha-synuclein aggregation can be directly accelerated by NP interaction provides a compelling direct mechanism but needs exploration for other disease-relevant proteins like amyloid-beta. A significant limitation across much of the mechanistic research is the heavy reliance on polystyrene particles, often pristine spheres, in animal models using relatively high exposure doses compared to estimated human environmental levels.

## 4 Observed neurological effects and associated outcomes

The intricate molecular and cellular disturbances instigated by microplastic (MP) and nanoplastic (NP) exposure, as detailed previously, manifest as a spectrum of observable adverse neurological effects and associated functional outcomes. Research across various experimental models, including rodents, zebrafish, and cell cultures, consistently points towards significant impacts on cognitive function, behavior, and neuropathology, including outcomes that bear resemblance to human neurodegenerative and neurodevelopmental disorders. Furthermore, emerging human epidemiological data, while currently limited, provides correlative links that underscore the potential relevance of these experimental findings to human brain health.

Cognitive impairment, particularly affecting learning and memory processes, is a frequently documented consequence of MP/NP exposure in animal models. Studies utilizing standard behavioral paradigms, such as the Morris water maze, have demonstrated impaired spatial learning and memory retention in mice following oral exposure to polystyrene MPs (Wang SW. et al., 2022). Similar deficits in learning and memory have been reported with polystyrene NP exposure, linked mechanistically to hippocampal dysfunction and neuroinflammation (Jin et al., 2022; Paing et al., 2024; Liu et al., 2025; Sharma et al., 2023). Cognitive decline associated with inhaled MPs has also been observed, potentially mediated via the lung-brain axis involving lipopolysaccharide signaling and microglial activation (Kang et al., 2024). Zebrafish models corroborate these findings, showing impaired learning and memory capacity following NP exposure, associated with markers of accelerated brain aging and oxidative damage (Zho et al., 2023). Even disruptions in circadian rhythm, linked to NP exposure via the gut-brain axis, have been shown to impair learning and memory abilities in mice (Kang et al., 2023). While direct causal evidence in humans is lacking, concerning correlations have been reported; higher exposure levels to plastic products have been associated with an increased risk of mild cognitive impairment (MCI) in older adults (Zhu et al., 2024), and post-mortem studies have revealed higher brain burdens of MNPs in individuals diagnosed with dementia (Nihart et al., 2025).

Beyond cognitive functions, MP/NP exposure induces a wide array of behavioral abnormalities across different species. Anxiety-like behaviors are commonly reported, evidenced by altered performance in tests like the elevated plus maze and open field test in rodents (Ma et al., 2024; Chen et al., 2023; Li et al., 2024; Su et al., 2025; Sharma et al., 2023; Shin et al., 2023), and increased thigmotaxis or erratic swimming patterns in zebrafish (Félix et al., 2023; Torres-Ruiz et al., 2023). Depressive-like behaviors have also been associated with NP exposure in mice (Ma et al., 2024; Su et al., 2025; Kuai et al., 2024). Locomotor activity is frequently affected, although the direction of change can vary; studies report both hyperactivity (Chen et al., 2020) and hypoactivity or lethargy (Torres-Ruiz et al., 2023; Sun et al., 2021; Jeong S. et al., 2022). Impaired swimming capacity in fish has been linked to underlying physiological toxicity (Liu et al., 2024). Critically, specific movement disorders resembling symptoms of PD, such as impaired motor skills, have been induced by MP/NP exposure in models incorporating alpha-synuclein pathology or direct dopaminergic neuron targeting (Liang et al., 2025; Bai et al., 2025; Xian et al., 2024). Disruptions to circadian rhythms also manifest as altered activity patterns (Sarasamma et al., 2020; Kang et al., 2023; Luan et al., 2023). Furthermore, MP/NP exposure has been shown to impair social behaviors, including reduced social novelty preference in mice (Ma et al., 2024; Shin et al., 2023; So et al., 2023) and altered shoaling or predator avoidance behaviors in fish (Chagas et al., 2021; Guimaraes et al., 2021; Rios-Fuster et al., 2021). Some studies explicitly report the induction of autism spectrum disorder (ASD)-like behaviors in mice following developmental or direct exposure (Xian et al., 2024; Zaheer et al., 2022).

Perhaps most concerning are the findings linking MP/NP exposure to pathological changes characteristic of specific human neurodegenerative diseases. Several studies provide evidence for a link to PD-like pathology. Exposure to polystyrene MPs and NPs has been shown to accelerate dopaminergic neuron degeneration and induce PD-like motor dysfunction in mouse models, particularly those involving the A53T alpha-synuclein mutation or direct intestinal exposure pathways (Liang et al., 2025; Bai et al., 2025; Liang et al., 2022; Liang et al., 2024c). This is underpinned by the mechanistic finding that MPs/NPs directly promote the formation of neurotoxic alpha-synuclein oligomers and aggregates (Huang et al., 2023; Ghosal et al., 2024). Alzheimer’s disease (AD)-like pathology is also implicated. Cognitive impairments induced by MPs/NPs (Wang GH. et al., 2024) are a hallmark of AD. Mechanistic studies show NP-induced Tau protein acetylation (Bai et al., 2024) and potential increases in Tau phosphorylation (Kim et al., 2025), both key features of AD neuropathology. While direct induction of amyloid-beta (Aβ) plaques by MPs/NPs is less clearly documented in the provided literature, studies have shown increased Aβ and P-tau deposition secondary to MP-induced gut dysbiosis and neuroinflammation via the gut-brain axis (Hu et al., 2023), suggesting an indirect contribution. Furthermore, accelerated brain aging markers observed with NP exposure could increase susceptibility to late-onset neurodegenerative conditions (Zho et al., 2023; Stefanova et al., 2024). Beyond specific PD and AD links, general neurodegenerative outcomes, such as significant neuronal death and brain tissue damage, have been reported following various MP/NP exposure scenarios (Vojnits et al., 2024; Yin et al., 2022a; Kim et al., 2025; Huang et al., 2025).

The neurological outcomes are particularly pronounced when exposure occurs during critical neurodevelopmental windows. Maternal exposure to MPs/NPs during gestation and/or lactation leads to a range of adverse effects in offspring brain development and function. Structural abnormalities reported include alterations in brain morphology, reduced cortical thickness, disordered neuronal migration, and changes in the structure of specific brain regions like the hippocampus and motor cortex (Harvey et al., 2023; Jeong B. et al., 2022; Tian et al., 2024). These structural changes are accompanied by functional deficits, such as impaired cognitive function and altered neurophysiological activity in later life (Jeong B. et al., 2022; Tian et al., 2024). Developmental exposure disrupts fundamental processes like neurogenesis and impairs synaptic development (Yang S. et al., 2023; Tian et al., 2024). Exposure can also affect the timing of developmental milestones (Harvey et al., 2023). Importantly, developmental MP/NP exposure has been directly linked to an increased risk of ASD-like behavioral phenotypes in offspring (Zaheer et al., 2022; Jeong B. et al., 2022). Epidemiological observations in children showing associations between urinary MP levels and increased behavioral problems (emotional, conduct, hyperactivity, peer issues) and decreased prosocial behavior provide a concerning, though correlational, parallel in humans (Dong et al., 2025).

A major strength of the current evidence is the mechanistic linkage provided by many studies - observed outcomes like memory impairment are often correlated with demonstrable pathological changes in relevant brain regions or disruptions in specific neurotransmitter systems. The consistency in reports of oxidative stress and neuroinflammation as underlying factors across different outcome measures also provides a coherent toxicological narrative. The specific finding that NPs can directly promote alpha-synuclein aggregation offers a particularly strong, molecular-level link to PD pathogenesis, moving beyond simple correlation.

However, challenges remain, primarily concerning extrapolation to human scenarios. While animal studies clearly show causal links between controlled exposures and neurological effects, human evidence remains largely correlational. Some direct evidence has established that these particles can access the human central nervous system; various polymer types, including polyethylene and polystyrene, have been identified in human cerebrospinal fluid, and MPs have been detected in post-mortem olfactory bulb tissue. Building on this, observational studies have begun to link exposure with adverse neurological outcomes. For instance, higher plastic exposure levels have been correlated with an increased risk of mild cognitive impairment in older adults, and greater brain burdens of MPs/NPs have been found in individuals with dementia. In children, urinary MP levels have been associated with a higher incidence of behavioral problems. However, it is critical to underscore that all current human evidence is observational and correlational. A definitive causal link between MP/NP exposure and human neurotoxicity has not yet been established.

Contradictions or nuances also exist within the literature. While many studies report significant behavioral or cognitive deficits, others find only subtle, transient, or even absent effects under specific conditions (So et al., 2023; Gaspar et al., 2023; Rafiee et al., 2018). Some biodegradable plastics, like certain PLA formulations, induced less overt locomotor damage compared to conventional plastics, although they still caused biochemical disturbances (Chagas et al., 2021). These variations likely reflect differences in particle type (polymer, size, shape, charge, aging status), exposure dose and duration, species or strain sensitivity, and the specific endpoints measured. The predominant focus on polystyrene spheres in many studies limits generalizability to the diverse array of plastic particles humans encounter. The effects of aged, irregularly shaped particles derived from environmental degradation, which likely constitute the bulk of human exposure, are comparatively understudied regarding neurological outcomes.

## 5 Factors influencing microplastic/nanoplastic neurotoxicity

The manifestation and severity of neurological effects resulting from microplastic (MP) and nanoplastic (NP) exposure are not uniform but are significantly modulated by a confluence of factors. Studies highlight particle size, shape, polymer type, surface properties (including charge and modifications like aging or the formation of a biomolecular corona), the “vector effect” of co-transported chemicals, and host characteristics such as age and sex as key modulators of neurotoxicity.

Particle characteristics are perhaps the most extensively studied determinants of MP/NP toxicity. Size plays a critical role, influencing biodistribution, cellular uptake, barrier penetration, and subsequent toxicological effects (Yang ZS. et al., 2022; Cho et al., 2024). As discussed regarding brain access, NPs generally exhibit greater potential to cross biological barriers like the BBB compared to MPs, often leading to more pronounced direct CNS effects (Hamed et al., 2022a; Hwang et al., 2022). However, the relationship between size and toxicity is not always linear across all endpoints or particle types. For instance, Ding JN (Ding et al., 2020) observed that mid-sized MPs (5 µm) elicited greater oxidative stress and metabolic disruption in red tilapia compared to both smaller NPs and larger MPs, suggesting a potential peak toxicity within a specific size range for certain effects. Different sized particles also induce distinct patterns of gene expression and metabolic pathway alterations (Lu et al., 2022; Xue et al., 2022). The polymer type inherently dictates the particle’s chemical properties, surface energy, and interaction potential, yet comparative neurotoxicity studies across different polymer types are relatively scarce in the provided literature, with a heavy focus on polystyrene (PS). Sheng et al. (2021) demonstrated polymer-specific differences in adsorbing co-contaminants, which indirectly influences combined toxicity. Surface properties, including charge and chemical modifications, are also critical. Positively charged PS-NPs were found to induce greater neurobehavioral impairment in zebrafish compared to negatively charged ones (Teng et al., 2022), and different surface modifications (e.g., amino, carboxyl) influence cellular internalization pathways and resultant toxicity profiles (Bai et al., 2024; Ma et al., 2024). Environmental aging significantly alters particle surfaces (e.g., increasing roughness, introducing oxygen-containing functional groups) and can enhance their toxic potential, including inflammatory responses, compared to pristine particles (Qian et al., 2025; Kim et al., 2023; Zhang et al., 2022).

The interaction of MPs/NPs with other environmental contaminants significantly influences their overall neurotoxic impact, often referred to as the “vector effect” or “Trojan Horse” effect. MPs/NPs can adsorb various pollutants, including heavy metals, pesticides, pharmaceuticals, persistent organic pollutants (POPs), and bacterial toxins like LPS, from the environment onto their surfaces (Zheng et al., 2024; Kang et al., 2024; Rainieri et al., 2018). This association can alter the transport, distribution, and bioavailability of the adsorbed chemicals within organisms (Trevisan et al., 2020). Co-exposure can lead to combined toxic effects that may be additive, synergistic, or antagonistic, depending on the specific plastic, contaminant, dose, and biological endpoint measured (Zhao KM. et al., 2023; Yang J. et al., 2023). For example, co-exposure of PS-NPs with 4-methylbenzylidene camphor intensified tissue accumulation and resulted in sex-specific neurotoxicity and reproductive disruption in zebrafish (Xian et al., 2024). Similarly, combined exposure to PP-MPs and DEHP induced synergistic neurotoxic effects in immature mice (Yang G. et al., 2023). Conversely, some studies report antagonistic interactions where MPs may reduce the bioavailability or toxicity of the co-pollutant, potentially by sequestering it or altering its metabolic pathway (Wang J. et al., 2022; Espinosa-Ruiz et al., 2023; Hanslik et al., 2022). The aging status of MPs can also modify these interactions, influencing sorption/desorption kinetics and the resulting combined toxicity (Huang et al., 2021).

Host-specific factors also play a crucial role in determining susceptibility to MP/NP neurotoxicity. Age is a significant modifier, with developmental stages being particularly vulnerable periods due to ongoing neurogenesis, synaptogenesis, and barrier maturation (So et al., 2023; Jeong B. et al., 2022; Tian et al., 2024; Yang DQ. et al., 2022). Maternal exposure during pregnancy and lactation can lead to distinct neurodevelopmental abnormalities and long-lasting behavioral consequences in offspring (Dibbon et al., 2024; Harvey et al., 2023; Kaur et al., 2025). Studies have also observed age-dependent differences in behavioral and immune responses to acute MP exposure in adult animals (Gaspar et al., 2023). Sex represents another important host factor, with several studies reporting sex-specific neurological and behavioral outcomes following MP/NP exposure (Xian et al., 2024; Jeong B. et al., 2022). The underlying reasons for these sex differences are likely complex, potentially involving hormonal influences on MP/NP metabolism, distribution, immune responses, or direct effects on sex-specific neural circuits, but require further investigation. Finally, species-specific differences in physiology, metabolism, and behavior undoubtedly influence MP/NP uptake, distribution, and toxic response, highlighting the need for caution when extrapolating findings across different animal models or to humans (Capó et al., 2022).

## 6 Potential mitigation strategies

While preventing environmental contamination remains the ultimate goal, identifying interventions capable of counteracting or ameliorating the neurotoxic effects induced by ingested or inhaled particles is essential for protecting brain health. The literature suggests several promising avenues, primarily focusing on dietary supplements, antioxidant therapies, modulation of the gut microbiota, and potentially targeting specific molecular pathways compromised by MP/NP exposure. These strategies aim to counteract key pathogenic mechanisms such as oxidative stress, neuroinflammation, mitochondrial dysfunction, and gut-brain axis disruption.

Dietary interventions and supplementation with specific natural products or antioxidants represent a frequently explored approach. Natural antioxidants have shown efficacy in experimental models. For instance, the antioxidant Vitamin E was demonstrated to reverse learning and memory deficits induced by polystyrene MPs in mice, correlating with its ability to combat oxidative stress (Wang SW. et al., 2022). Similarly, N-acetylcysteine (NAC), another potent antioxidant, showed protective effects against PS-NP-induced cytotoxicity and autophagy in neuronal cell lines (Tang et al., 2022). Functional food components like Camellia pollen demonstrated significant mitigation of amino-modified polystyrene NP-induced neurotoxicity, including protection against BBB disruption and neuronal apoptosis (Bai et al., 2024). Epigallocatechin-3-gallate (EGCG), the major polyphenol in green tea, was found to prevent PS-MP-induced anxiety-like behavior in mice, potentially by modulating the gut-brain axis and inhibiting neuroinflammation (Yang JZ. et al., 2023). Other natural compounds, including betaine (Kamel et al., 2025) and dietary additives used in aquaculture like Chlorella, citric acid, and lycopene (Hamed et al., 2022a), have also shown promise in alleviating MP-induced neurotoxicity and related biochemical disturbances in animal models. Melatonin, known for its antioxidant and chronobiotic properties, has emerged as another potential mitigator, shown to alleviate PS-NP neurotoxicity by regulating circadian rhythm genes (Kang et al., 2023) and counteracting excessive mitophagy and dopaminergic neuron death (Huang et al., 2023). Additionally, hydrogen-rich water (HRW) demonstrated efficacy in mitigating MP-induced toxicity in fish, possibly through redox modulation (Atamanalp et al., 2023).

Modulation of the gut microbiota and maintaining gut health appear as particularly important strategies, given the strong mechanistic links established via the gut-brain axis. Several studies suggest that targeting the gut microbiome could be a novel therapeutic approach for MP/NP-induced neurological harm (Shi et al., 2024; Pan et al., 2024). Specific interventions have shown promise: the administration of probiotics, such as *Lactobacillus* plantarum DP189, in combination with prebiotics like galactooligosaccharides (GOS), effectively alleviated cognitive dysfunction induced by polyethylene MPs in mice, associated with improved gut barrier integrity and reduced neuroinflammation (Wang J. et al., 2024). Probiotics were also effective, alongside melatonin, in mitigating PS-NP neurotoxicity linked to circadian rhythm disruption (Kang et al., 2023). Fecal microbiota transplantation (FMT) has also been shown to reverse neurological impairment caused by combined MP and doxycycline exposure by restoring gut microbial balance (Sun et al., 2024). Furthermore, bile acid supplementation demonstrated significant alleviation of neurotoxicity induced by aged polylactic acid MPs in zebrafish, likely acting via the gut-brain axis (Qian et al., 2025). These findings collectively underscore the critical role of maintaining intestinal homeostasis-encompassing barrier function and microbial balance-in potentially reducing the neurological consequences of MP/NP exposure (Liang et al., 2024b).

Beyond dietary and microbial strategies, interventions targeting specific molecular pathways implicated in MP/NP neurotoxicity are being explored. Specific activation of the astrocyte glutamate transporter EAAT2 was shown to reverse anxiety and depressive-like behaviors induced by PS-NPs in mice, highlighting glutamate homeostasis as a potential target (Su et al., 2025). Similarly, targeting the ErbB4 receptor pathway using a small molecule agonist (E4A) ameliorated neuronal deficits and neuroinflammation in a mouse model of PS-MP exposure, suggesting another potential pharmacological target (Liu et al., 2025). While these molecularly targeted approaches are currently preclinical, they indicate the potential for developing more specific therapies based on a deeper understanding of the downstream signaling cascades disrupted by MPs/NPs.

## 7 Conclusion and future perspectives

The collective evidence reviewed he rein strongly indicates that MPs and NPs represent a tangible, emerging threat to brain health. These ubiquitous particles possess the capacity to breach critical biological barriers, including the BBB and the placental barrier, enabling their access to the CNS and the developing fetal brain. Once present, MPs and NPs are far from inert; they initiate a complex interplay of detrimental processes, including oxidative stress, persistent neuroinflammation involving microglia and astrocytes, mitochondrial dysfunction leading to energy deficits, disruption of crucial neurotransmitter systems, and direct neuronal damage. Critically, NPs have been shown to promote the aggregation of proteins implicated in neurodegenerative conditions like PD. These mechanistic disturbances translate into observable adverse outcomes in experimental models, ranging from cognitive impairments in learning and memory to a spectrum of behavioral abnormalities including anxiety, depression, and social deficits, as well as pathologies resembling human neurodegenerative and neurodevelopmental disorders. While the influence of particle characteristics, co-exposures, and host factors adds layers of complexity, and some mechanistic details like the precise role of the biomolecular corona remain contested, the overall trajectory of findings points towards a significant neurotoxic potential. However, the current reliance on animal models, often using high doses of pristine particles, necessitates caution in directly extrapolating these findings to quantitative human risk assessment under conditions of chronic, low-level environmental exposure to diverse plastic mixtures.

Moving forward, research must prioritize bridging this critical translational gap. A paramount future direction is to elucidate the effects of environmentally realistic exposures on human brain health. This requires developing robust methods for quantifying human internal exposure to various MPs/NPs and correlating these internal doses with neurological endpoints in well-designed epidemiological studies, focusing on both neurodevelopmental outcomes in children and cognitive decline or neurodegenerative disease risk in adults. Complementing this, advanced human-relevant *in vitro* models, such as sophisticated BBB-on-a-chip systems and brain organoids, should be employed to study mechanisms using lower, chronic doses of environmentally aged, mixed-polymer particles and their leachates. A second crucial direction involves dissecting the relative contributions of direct versus indirect neurotoxic mechanisms under chronic, low-dose conditions. It is imperative to understand whether the primary driver of neurological harm stems from the physical presence and direct interaction of particles within the brain parenchyma, or from systemic inflammation and metabolic disruption originating in the gut or lungs and communicated via the gut-brain or lung-brain axes. This necessitates studies capable of differentiating these pathways, perhaps by comparing systemic versus direct CNS administration or using advanced imaging and biomarker techniques to track both particle translocation and systemic signalling over long exposure periods relevant to human lifespans.

Future investigations should also pivot to the nano-scale to address critical methodological and mechanistic gaps. A key priority is the development of novel intracellular tracking technologies, such as hyperspectral imaging and advanced fluorescence or Raman microscopy, to visualize and quantify the subcellular fate of NPs in real-time within neurons and glia. This would allow researchers to directly observe processes like lysosomal escape and nuclear translocation. Furthermore, studies must move beyond pristine polystyrene spheres to investigate how environmental aging, irregular shapes, and diverse polymer chemistries influence the formation and composition of the biomolecular corona, which ultimately dictates the neurotoxic potential of these particles. Understanding the specific molecular interactions at this bio-nano interface could reveal why certain NPs accelerate the aggregation of proteins like alpha-synuclein and may uncover similar effects on other key proteins like amyloid-beta and Tau. Paradoxically, a deeper mechanistic understanding of how NPs breach the blood-brain barrier and interact with cellular machinery could even pave the way for designing novel polymer-based nanoparticles as advanced drug delivery systems for treating the very neurodegenerative diseases they are suspected of exacerbating.

Addressing these key areas will be essential not only for accurately assessing the public health risks posed by microplastic pollution but also for guiding effective preventative policies and developing potential interventions grounded in a solid understanding of human-relevant pathophysiology.
